# Spatial Compartmentalization of the Microbiome between the Lumen and Crypts Is Lost in the Murine Cecum following the Process of Surgery, Including Overnight Fasting and Exposure to Antibiotics

**DOI:** 10.1128/mSystems.00377-20

**Published:** 2020-06-09

**Authors:** Alexander Zaborin, Beatriz Penalver Bernabe, Robert Keskey, Naseer Sangwan, Sanjiv Hyoju, Neil Gottel, Jack A. Gilbert, Olga Zaborina, John C. Alverdy

**Affiliations:** a Department of Surgery, University of Chicago, Chicago, Illinois, USA; b Department of Bioengineering, University of Illinois at Chicago, Chicago, Illinois, USA; c Microbial Composition and Analytics Core, Department of Cardiovascular and Metabolic Sciences, Cleveland Clinic, Cleveland, Ohio, USA; d Department of Pediatrics, University of California San Diego, La Jolla, California, USA; Teagasc Food Research Centre

**Keywords:** 16S rRNA, butyrate, cecal crypts, hypoxia gradient, *Mucispirillum schaedleri*, murine cecum microbiome, shotgun sequencing, spatial compartmentalization, surgery

## Abstract

The cecum is a unique region in the mammalian intestinal tract in which the microbiome is localized to two compartments, the lumen and the crypts. The microbiome within crypts is particularly important as it is in direct contact with lining epithelial cells including stem cells. Here, we analyzed the microbiome in cecum of mice using multiple techniques including metagenomics. The lumen microbiome comprised *Firmicutes* and *Bacteroidetes* whereas the crypts were dominated by *Proteobacteria* and *Deferribacteres*, and the mucus comprised a mixture of these 4 phyla. The lumen microbial functional potential comprised mainly carbon metabolism, while the crypt microbiome was enriched for genes encoding stress resistance. In order to determine how this structure, assembly, and function are altered under provocative conditions, we exposed mice to overnight starvation (S), antibiotics (A), and a major surgical injury (partial hepatectomy [H]), as occurs with major surgery in humans. We have previously demonstrated that the combined effect of this “SAH” treatment leads to a major disturbance of the cecal microbiota at the bottom of crypts in a manner that disrupts crypt cell homeostasis. Here, we applied the SAH conditions and observed a loss of compartmentalization in both composition and function of the cecal microbiome associated with major shifts in local physicochemical cues including decrease of hypoxia, increase of pH, and loss of butyrate production. Taken together, these studies demonstrated a defined order, structure, and function of the cecal microbiome that can be disrupted under provocative conditions such as major surgery and its attendant exposures.

**IMPORTANCE** The proximal colon and cecum are two intestinal regions in which the microbiome localizes to two spatially distinct compartments, the lumen and crypts. The differences in composition and function of luminal and crypt microbiome in the cecum and the effect of physiological stress on their compartmentalization remain poorly characterized. Here, we characterized the composition and function of the lumen-, mucus-, and crypt-associated microbiome in the cecum of mice. We observed a highly ordered microbial architecture within the cecum whose assembly and function become markedly disrupted when provoked by physiological stress such as surgery and its attendant preoperative treatments (i.e., overnight fasting and antibiotics). Major shifts in local physicochemical cues including a decrease in hypoxia levels, an increase in pH, and a loss of butyrate production were associated with the loss of compositional and functional compartmentalization of the cecal microbiome.

## Abstract (precis)

The proximal colon and cecum are two intestinal regions in which the microbiome localizes to two spatially distinct compartments, the lumen and crypts. The differences in composition and function of luminal and crypt microbiome in the cecum and the effect of physiological stress on their compartmentalization remain poorly characterized. Here, we characterized the composition and function of the lumen-, mucus-, and crypt-associated microbiome in the cecum of mice. We observed a highly ordered microbial architecture within the cecum whose assembly and function become markedly disrupted when provoked by physiological stress such as surgery and its attendant preoperative treatments (i.e., overnight fasting and antibiotics). Major shifts in local physicochemical cues including a decrease in hypoxia levels, an increase in pH, and a loss of butyrate production were associated with the loss of compositional and functional compartmentalization of the cecal microbiome.

## INTRODUCTION

Renewed interest in the mammalian cecum and appendix has emerged given their unique anatomic conformation, their exclusion from the direct fecal stream, the rich microbiome they harbor, and their impact on host immune function (1, 2). When the cecum is surgically removed from mice, the intestinal microbiome is changed, immune function is impaired, butyrate levels are decreased, and there is a loss of colonization resistance to exogenous pathogens (2). Our group has been interested in the structure, membership, and function of the cecal microbiome within both the lumen and crypts given that the lumen microbiome is frequently sampled and considered representative of the global gut microbiome. Prior work from our lab has established that provocative conditions such as *s*tarvation, *a*ntibiotics, and a partial *h*epatectomy (SAH) can disturb the cecal crypt microbiome such that its underlying stem cell population is impaired.

The murine cecum is a well-recognized intestinal organ that harbors a specialized microbiome involved in the fermentation of dietary fiber to produce short-chain fatty acids (3). An intriguing feature of murine cecum and proximal colon microbiome is how it is spatially configured to compartmentalize into luminal and crypt microbiome with the crypt microbiome lying in close proximity to stem cells (4). Studies to date have characterized the proximal colon, but not the cecum proper, via genetic techniques to identify crypt microbiome (4). Results from these studies using laser capture microdissection (LCM) and 16S rRNA gene amplicon sequencing (4) identified Acinetobacter as a major member of the crypt community microbiome (4).

Although the cecal crypt microbiome has not been characterized via genetic sequencing *per se*, spiral-shaped bacteria have been observed to dominate in the cecal crypts of rats as judged by light and transmission electron microscopy (TEM) (5, 6). The observed morphotype was distinct from Acinetobacter, the genus previously reported to dominate the crypts of mice in the proximal colon. This discrepancy may be attributed to the use of different rodents (rat or mouse) and/or specific regions of the colon (proximal colon versus cecum). In addition, region-specific microbiomes in the cecum (i.e., the tip being furthest away from the fecal stream versus the base [see Fig. S1 in the supplemental material]) have not been previously characterized. As such, there is incomplete knowledge regarding the spatial and regional distribution of the microbiome in the cecum.

10.1128/mSystems.00377-20.1 FIG S1 Image of mouse cecum defining the “tip” and “base.” Download FIG S1, PDF file, 0.1 MB. Copyright © 2020 Zaborin et al. 2020 Zaborin et al. This content is distributed under the terms of the Creative Commons Attribution 4.0 International license.

Prior work from our group has demonstrated the essential role of the crypt microbiome to maintain stem cell homeostasis as reflected by the molecular markers LGR5 and Ki67 when provocative conditions are imposed on mice, such as the routine process of major surgery, which is invariably accompanied by overnight fasting and antibiotic exposure (7). Prior work from our laboratory indicated that the combined effects of overnight starvation, antibiotics, and surgery (SAH) were required to disrupt the cecal crypt microbiome while individually none alone was sufficient (7). Therefore, the aim of this study was to define the composition and metabolic potential of the cecal crypt microbiome in untreated mice at two locations in the cecum proper, the tip and the base. Mice then were exposed to SAH conditions to determine how these combined provocative stimuli altered baseline conditions.

## RESULTS

### Crypt microbial composition is significantly distinct from lumen composition.

**FIG 1:**
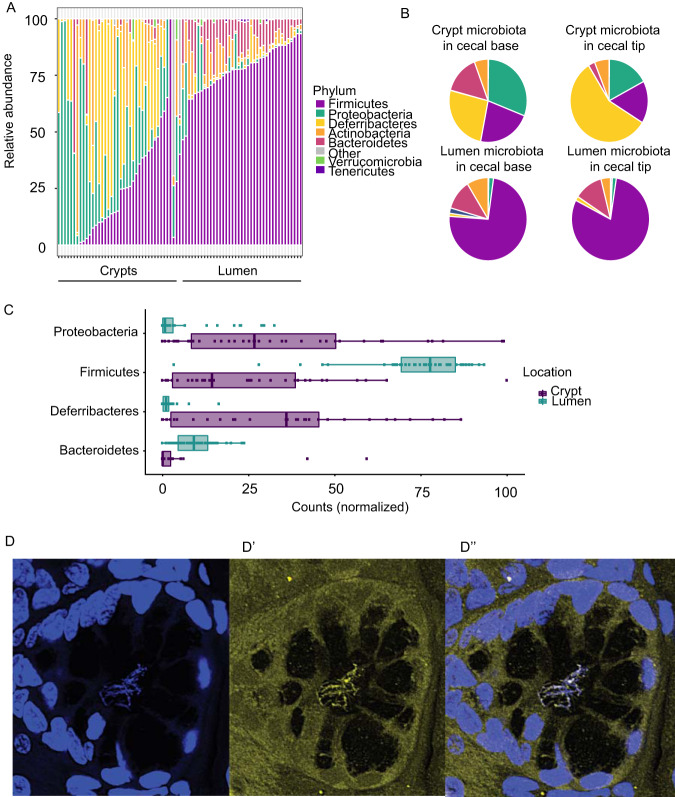
Crypt microbiota in the cecum are distinct from those in the lumen in untreated mice. (A) Taxonomic profile of crypt and luminal microbiota in individual mice. (B) Integrated taxonomic profile of crypt and luminal microbiota at the cecal tip and base. (C) Quantitative analysis correcting for batch effect and cecum proximity (FDR-corrected *P* < 0.001). *Bacteroidetes* and *Firmicutes* were more abundant in the lumen samples (FDR-corrected *P* < 0.001). *Proteobacteria* and *Deferribacteres* were more abundant in crypt samples (FDR-corrected *P* < 0.001 and 0.04, respectively). (D to Dʺ) FISH analysis to identify *Mucispirillum* in crypts. (D) DAPI staining for DNA labeling. (D′) Yellow fluorescent image to visualize *Mucispirillum* ATO550. (Dʺ) Converged image.

From a total of 102 samples, 34 environmental controls, and 6 kit controls, 2,898,381 16S rRNA sequence reads (average 29,780; minimum 2,880, maximum 93,8083 sequence reads per sample) remained after filtering and removing low-quality samples and chimeric sequences. These 16S rRNA sequence reads comprised 3,841 exact sequence variants (ESVs), of which 11.6% were present in the environmental control samples. The environmental control analysis revealed that the family *Enterobacteriaceae* and the genus Acinetobacter were the dominant contaminants in each sequence run (see Fig. S4 and Table S2 in the supplemental material). Of note, Acinetobacter was identified as a contaminant genus by others (8, 9). After subtraction of environmental control sequences, we observed a statistically significant variability between extraction batches (false-discovery rate [FDR]-corrected *P* < 0.005) but not between mice from the same batch. The relative position within the cecum (i.e., regional changes; base versus tip) did not contribute to the differences between samples (Table S3). Therefore, all subsequent statistical estimations were controlled by the corresponding extraction batch. Results demonstrated that the crypts were colonized with bacteria distinct from those in the lumen. While *Proteobacteria* and *Deferribacteres* were the most common phyla in the crypt samples, the lumen was predominantly colonized by *Firmicutes* (Fig. 1A to C). Although some compositional trends were present between the regional specific localizations within the cecal crypts, i.e., more *Deferribacteres* at the tip versus more *Proteobacteria* at the base (Fig. 1B), these differences did not reach statistical significance.

The *Deferribacteres* phylum was mapped only to Mucispirillum schaedleri, which is an anaerobic spiral-shaped bacterium previously shown to colonize the mucus of the rodent gastrointestinal tract (10). Using fluorescence *in situ* hybridization (FISH) analysis with probes specific to *Mucispirillum*, we confirmed its presence inside the microbial bundles in crypts (Fig. 1D to Dʺ).

**FIG 2:**
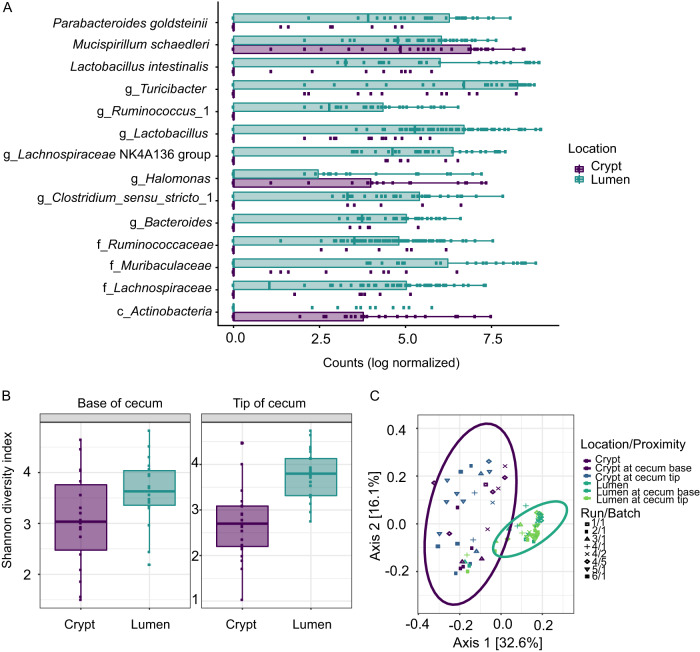
Structural analysis of cecal and crypt microbiota. (A) Analysis of amplicon sequence variants (ESVs) corrected for batch effect and cecum proximity. FDR-corrected *P* value of <0.005. (B) The analysis of α-diversity of cryptal and luminal microbiota at the base and at the tip of cecum. Lumen samples have a larger number of distinct ESVs than crypt samples and are more diverse (*P* < 0.05). No differences were observed between the locations in the cecum from which the samples were collected, e.g., cecum tip versus cecum base (*P* > 0.1). (C) The analysis of β-diversity measured by weighted nonnormalized UniFrac revealed significant differences between the lumen and the crypt samples (*P* < 0.001).

At a more granular level, generalized linear models (GLM) confirmed enrichment of *M. schaedleri* in the crypts compared to the lumen (Fig. 2A). Crypts were also enriched in ESVs annotated to *Halomonas* and *Actinobacteria*. The lumen samples had a greater proportion of ESVs annotated to Parabacteroides goldsteinii, Lactobacillus intestinalis, and *Turicibacter* (FDR-corrected *P* < 0.001), which were absent in crypts (Fig. 2A). Bacterial α-diversity (Shannon index) was greater in the lumen than in the crypts (*P* < 0.05) irrespective of cecal anatomic geography (Fig. 2B). The β-diversity (weighted UniFrac) was significantly different between crypt- and lumen-associated microbial communities (*P* < 0.001), and the β-diversity distance dissimilarity between crypt samples from different batches of mice was much greater than that between lumen samples (*P* < 0.001) (Fig. 2C).

**FIG 3:**
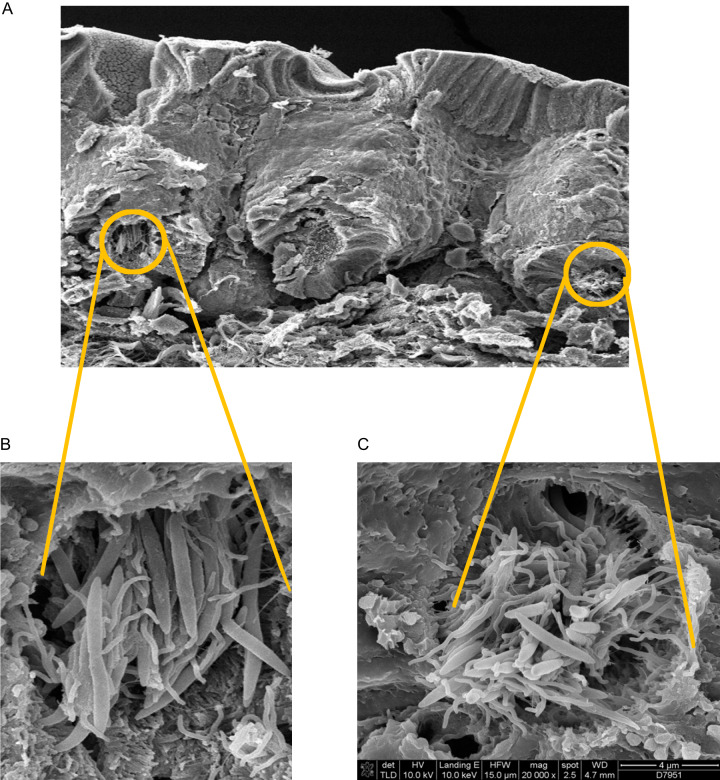
Scanning electron microscopy images of crypt microbiota. (A) SEM image representing two neighboring crypts. (B) Left-side crypt mainly contains bacteria of two morphological phenotypes with the dominance of long rods. (C) Right-side crypt contains bacteria of three morphological phenotypes representing by curved rods, long rods, and short rods.

The ESV composition was highly correlated within batches in the mouse-to-mouse lumen comparison but not in crypts (Fig. S5), suggesting a higher variability of microbiota composition between crypts. Furthermore, by scanning electron microscopy (SEM) analysis, we observed differences in bacterial morphology across neighboring crypts (Fig. 3A), indicating the microbiota composition variability at the crypt-to-crypt level. For instance, some crypts were colonized by long rod and spiral-shaped bacteria (Fig. 3B) while in neighboring crypts, domination by spiral-shaped bacteria and the presence of short rods were observed (Fig. 3C). However, in general, spiral-shaped and long rod bacteria were the most prevalent.

### Mucus-associated microbiota share similarities with the crypt and lumen microbiota.

**FIG 4:**
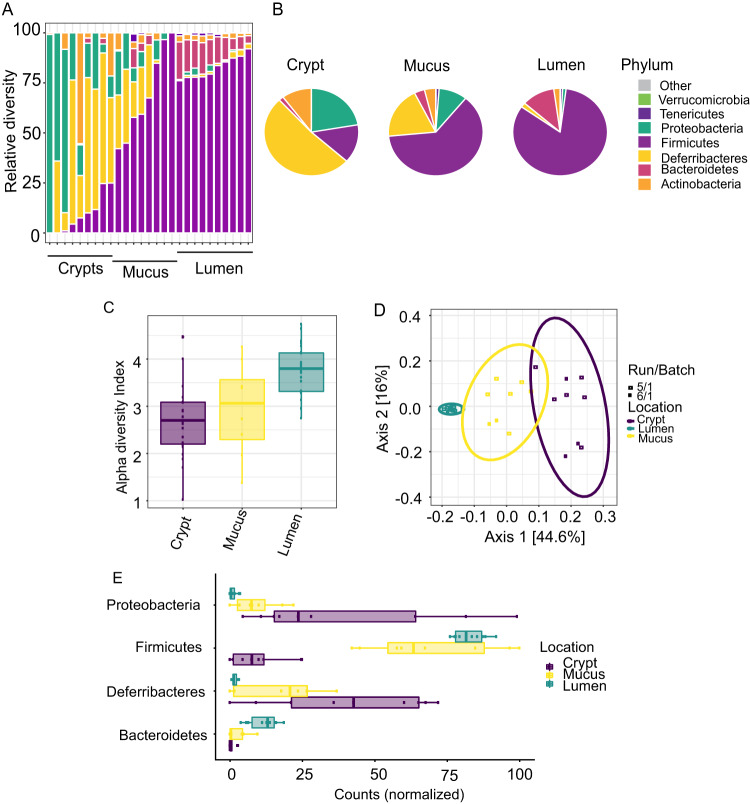
Comparative analysis of lumen, mucus, and crypt microbiota. (A) Taxonomic profile of crypt and luminal microbiota in individual mice. (B) Integrated taxonomic profile of crypt and luminal microbiota. (C) Analysis of α-diversity of cryptal, mucus, and luminal microbiota. (D) β-Diversity measured by weighted UniFrac. (E) Phylum abundance differences between the crypt, mucus, and lumen samples (FDR corrected for batch effect, *P* < 0.001).

In a separate set of experiments (*n* = 10 mice), crypt, mucus, and luminal contents from the cecum were extracted by LCM (Fig. S2) and characterized by 16S rRNA gene amplicon sequencing. Results demonstrated that mucus-associated microbiota were dominated by *Firmicutes* and contained *Bacteroidetes*, characteristic of the lumen microbiota. Similarly, mucus contained *Proteobacteria* and *Deferribacteres*, characteristics of the crypt microbiota (Fig. 4A and B). Diversity (α-diversity [Fig. 4C] and β-diversity [Fig. 4D]) is summarized in Fig. 4C to E. The proportion of ESVs is summarized in Fig. 4E. Many similarities can be seen in the measured parameters in mucus that appear to be shared between the lumen and crypts.

10.1128/mSystems.00377-20.2 FIG S2 Extraction of cecal contents by LCM. (A) Histology of the cecum tissue prior to laser capture microdissection. (B) After extraction of luminal content. (C) After extraction of mucus. (D) After extraction of crypt contents. Download FIG S2, PDF file, 0.5 MB. Copyright © 2020 Zaborin et al. 2020 Zaborin et al. This content is distributed under the terms of the Creative Commons Attribution 4.0 International license.

### Mucispirillum schaedleri is a keystone member of the cecal microbiota.

**FIG 5:**
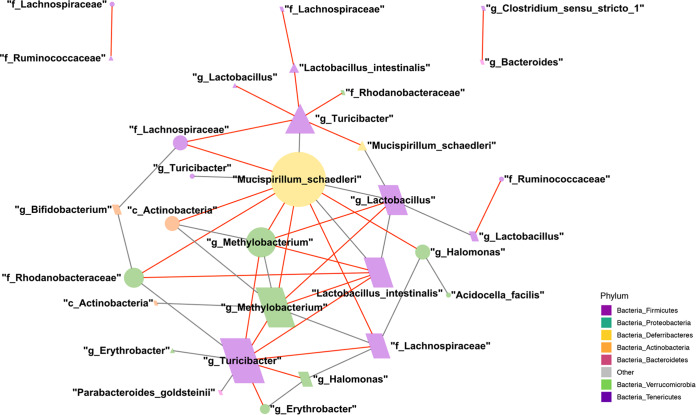
Coabundance networks between the significant ESVs in the lumen, crypt, and mucus. Negative correlations between the abundance of two different ESVs are depicted with red edges.

Analysis of the ESV coabundance networks between the lumen, crypt, and mucus (Fig. 5) showed that *M. schaedleri* was a hub node (vertex importance) that controlled connections between different areas of the coabundance as measured by betweenness (connection level between multiple communities within the network) and by degree of centrality (the number of edges that connect a node to the rest of the nodes). These results indicated that *M. schaedleri* might be an important species in the overall network stability. Although not conclusive, these observations imply that *M. schaedleri* may influence the relative abundance of many different taxa. For example, *M. schaedleri* relative abundance negatively correlated with the relative abundances of *Methylobacterium*, *Lachnospiraceae*, and *Actinobacteria*, while it was positively associated with *Lactobacillus* relative abundance. In the mucus, we observed that the relative abundance of *M. schaedleri* was positively correlated with that of the genus *Turicibacter*.

### Predicted metabolic analysis and shotgun metagenomics suggest that microbial metabolism in the lumen is associated with nutrient digestion while the crypt microbiome is associated with stress-related responses.

**FIG 6:**
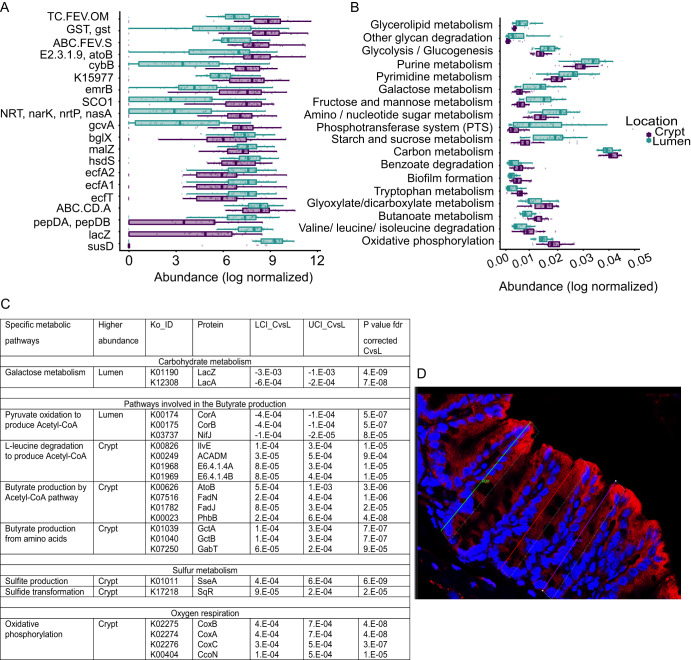
Functional metabolic potential of lumen and crypt microbiome. (A) Top 10 luminal and crypt genes that encode enzymes. FDR-corrected *P* < 0.001. (B) Potential metabolic pathways. FDR-corrected *P* < 0.001. (C) Predicted KEGG enzymes. CoA, coenzyme A. (D) Hypoxia immunochemistry staining of cecal crypts.

The predicted functional potential for all samples was obtained through Piphillin (11). The predicted genes that statistically significantly differed between the crypt and lumen comprised starch-binding outer membrane lipoprotein gene *susD* or β-galactosidase gene *lacZ*, which cleaves lactose into monosaccharides in lumen, while glutathione-disulfide reductase (*gsr*) and glutathione *S*-transferase (*gst*) were enriched in the crypts. Lumen-enriched predicted genes were associated with digestive functions, while crypt predicted genes were associated with oxidative and iron limitation stress response (Fig. 6A). In agreement with the top predicted genes in each cecal compartment, the predicted metabolic pathways confirmed that the oxidative phosphorylation and glyoxylate and dicarboxylate metabolic pathways were enriched in the crypts, while carbohydrate metabolism, an essential bacterial pathway for carbon source and energy production, and glycerolipid metabolism, critical for homeostasis of cellular lipid stores and membranes, were enriched in the lumen (Fig. 6B and C). In order to confirm that the crypt microbiome was indeed exposed to increased oxygen concentrations (i.e., less hypoxia in the normally hypoxic environment of the crypts), we performed immunohistochemical staining of cecal crypts for hypoxia. A strong inverse hypoxia gradient from the top to the bottom of the crypts suggests that those microbes at the bottom of the crypts were indeed exposed to elevated oxygen concentrations (Fig. 6D).

**FIG 7:**
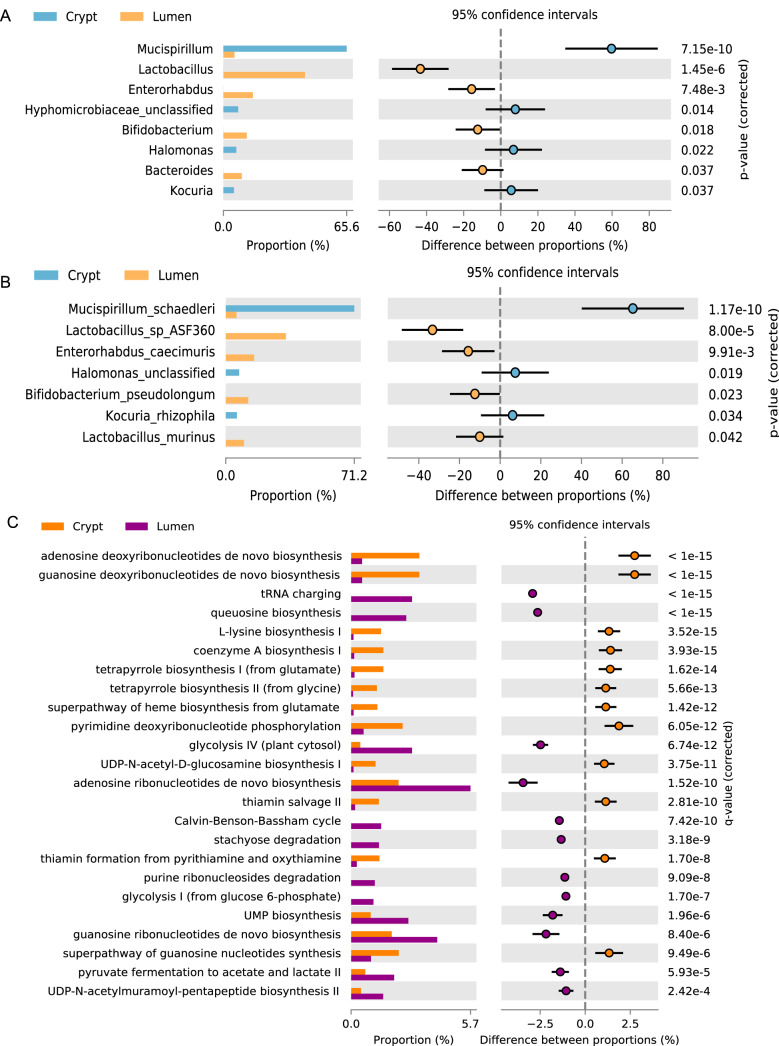
Metagenomic analysis of crypt and lumen microbiome. (A) Proportion (%) of genera. (B) Proportion (%) of species. (C) Proportion (%) of metabolic pathways.

Shotgun sequencing of DNA isolated from the cryptal and luminal contents extracted by LCM was used for metagenomics analysis. The average sequencing depth (after quality filtering) was 13,171,818 reads per sample (4.09 Gbp/sample, maximum = 27,940,496 and minimum of 694,208). According to Metaphlan analysis, the maximum number of species reported in crypt samples was 9, for which the current sequencing depth seems appropriate for the downstream analysis (12). Metagenomics analysis confirmed that Mucispirillum schaedleri was the dominant species in the crypt compartment followed by *Halomonas* (Fig. 7A and B). *Firmicutes* (*Lactobacillus* ASF360 and Lactobacillus murinus), *Actinobacteria* (Bifidobacterium pseudolongum and Enterorhabdus caecimuris), and *Bacteroidetes* (*Bacteroides*) (Fig. 7A and B) were more prevalent in lumen, again confirming 16S rRNA results. Metagenome functional analysis revealed that *de novo* purine biosynthesis was enriched in the crypt microbiome, suggesting a limited availability of nucleotide precursors in the crypt compartment (Fig. 7C). The crypt microbiome was enriched for l-lysine, tetrapyrroles, heme, and coenzyme A biosynthesis. Given that lysine biosynthesis plays an important role in protecting cells under oxidative stress (13, 14), the antioxidant function of coenzyme A (15), and the important role of tetrapyrroles and heme biosynthesis in the oxidative stress response (16, 17), functional metabolic analysis of the shotgun sequences confirmed the oxidative stress response phenotype in the crypt microbiome. In contrast, glycolysis, tetrasaccharide stachyose degradation, and pyruvate fermentation (Fig. 7C) in the luminal microbiome functional phenotype confirmed the digestive/fermentative phenotype suggested by 16S rRNA Piphillin predictions.

### SAH treatment disrupts compartmentalization of the cecal microbiota.

**FIG 8:**
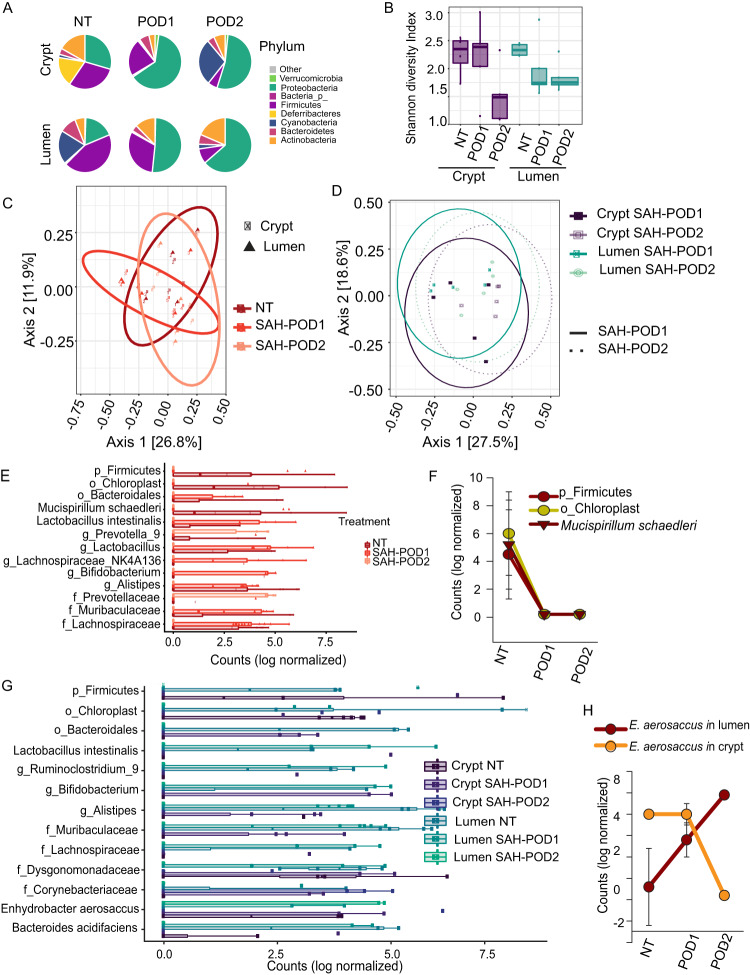
Loss of spatial compartmentalization of the cecal microbiota following SAH treatment. (A) Phylum abundance (FDR-corrected *P* value < 0.05). (B) Analysis of α-diversity of nontreated (NT), postsurgery day 1 (POD1), and POD2 cecal microbiota. (C) Analysis of β-diversity measured by weighted UniFrac of NT, POD1, and POD2 cecal microbiota. (D) Analysis of β-diversity measured by weighted UniFrac of POD1 and POD2 crypt and luminal microbiota. (E) Exact sequence variants (ESVs) between untreated (NT), 1-day postsurgery (POD1), and 2-day postsurgery (POD2) microbiota. FDR-corrected *P* value < 0.001. (F) Changes in relative abundance of stress-sensitive bacteria. FDR-corrected *P* value < 0.001. (G) ESVs between the NT, POD1, and POD2 groups in crypts and lumen demonstrating relocation of bacteria during surgical stress. (H) Relocation of Enhydrobacter aerosaccus from crypt to lumen with further propagation in lumen. FDR-corrected *P* value < 0.001. Crypt and luminal samples from cecal tip and base were combined into crypt and lumen groups.

Previous studies using the present mouse model of preoperative overnight starvation (S), exposure to a single dose of antibiotics (A), and major surgery with a 30% partial hepatectomy (H) (i.e., SAH) demonstrated significant disruption of the cecal crypt microbiota (7) in conjunction with disruption of crypt stem cell homeostasis. In the present study, we extended these observations and performed 16S rRNA gene amplicon sequence analysis of the luminal and cryptal microbiota collected at the first postoperative day (POD1) and on the second postoperative day (POD2) (Table S1). Results demonstrated that *Proteobacteria* became the dominating phylum at POD1 and POD2 in both the crypt and lumen microbiota while *Deferribacteres* became nondetectable after surgery (Fig. 8A). On the second day postsurgery (POD2), *Firmicutes* also significantly decreased (Fig. 8A). Microbial richness measured by the Shannon index dropped at POD2 in both compartments (Fig. 8B) with the composition significantly changed at POD1 and further at POD2 (Fig. 8C). Compositional differences between crypt and lumen microbiota at both POD1 and POD2 (Fig. 8D) were no longer observed, demonstrating that major surgery resulted in loss of compartmentalization of the cecal microbiota. ESV analysis of the overall cecal microbiota (combining crypt and lumen) revealed that *M. schaedleri* was no longer detectable after surgery (Fig. 8E). Additionally, an unclassified *Firmicutes* ESV and an unclassified *Cyanobacteriia Chloroplast* (order) ESV were among those unable to persist in SAH-treated mice and disappeared on POD1 (Fig. 8F). Moreover, ESVs such as proteobacterium *Enhydrobacter aerosaccus*, an unclassified *Firmicutes* ESV, and an unclassified *Cyanobacteriia Chloroplast* (order) ESV became detectable in the lumen samples (Fig. 8G). Importantly, only the crypt-associated *E. aerosaccus* was able to persist and became the dominating bacterium in the lumen at POD2 (Fig. 8H). None of the bacteria that relocated from the lumen to the crypts (i.e., unclassified *Bacteroidales*, *Bifidobacterium*, *Alistipes*, *Muribaculaceae*, and *Corynebacteriaceae* ESVs) were able to survive. Some lumen-associated ESVs such as Lactobacillus intestinalis, unclassified *Ruminiclostridium*, and *Lachnospiraceae* ESVs gradually disappeared from the lumen (Fig. 8G). Taken together, these data indicate that perturbations in the cecal microbiota following SAH treatment are characterized by transient relocations of bacteria to the regions where they are not able to survive and propagate, thus permissively allowing “beneficiary” species such as representatives of *Proteobacteria* to bloom.

10.1128/mSystems.00377-20.6 TABLE S1 Number of mice and samples used in the current project. NT, nontreated mice; SAHPOD1, mice subjected to preoperative starvation (S) (14 h), one dose of antibiotic treatment (A) (intramuscular injection of cefoxitin at a concentration of 25 mg/kg into the left thigh 30 min prior to surgery), and surgery of 30% left lobe hepatectomy (H) performed via a midline abdominal incision using electrocautery (an average surgical procedure time of 20 min). Mice sacrificed after 24 h after surgery (POD1). SAHPOD2, mice exposed to the same procedure as above and sacrificed after 48 h after surgery (POD2). Control samples represent environmental controls (no DNA added) that were subsequently processed along the cryptal, mucus, and luminal samples through the laser capture microdissection procedure, DNA isolation, and sequencing. *, mice used to study the effect of SAH treatment. Download Table S1, DOCX file, 0.01 MB. Copyright © 2020 Zaborin et al. 2020 Zaborin et al. This content is distributed under the terms of the Creative Commons Attribution 4.0 International license.

10.1128/mSystems.00377-20.7 TABLE S2 Total counts of ESVs that mapped to the genus Acinetobacter in environmental and mouse samples. Download Table S2, XLSX file, 0.04 MB. Copyright © 2020 Zaborin et al. 2020 Zaborin et al. This content is distributed under the terms of the Creative Commons Attribution 4.0 International license.

10.1128/mSystems.00377-20.8 TABLE S3 Identification of variabilities in 16S rRNA sequencing. All the PERMANOVA calculations have been blocked by the run and batch in which the samples were sequenced and extracted and mouse and location when appropriate. False-discovery rate (FDR) was employed to correct for multiple comparisons. Download Table S3, DOCX file, 0.03 MB. Copyright © 2020 Zaborin et al. 2020 Zaborin et al. This content is distributed under the terms of the Creative Commons Attribution 4.0 International license.

### The cecal microenvironment following SAH treatment is characterized by elevated oxygen concentrations at the top and bottom of the crypts, loss of butyrate, and alkalinization of the lumen.

**FIG 9:**
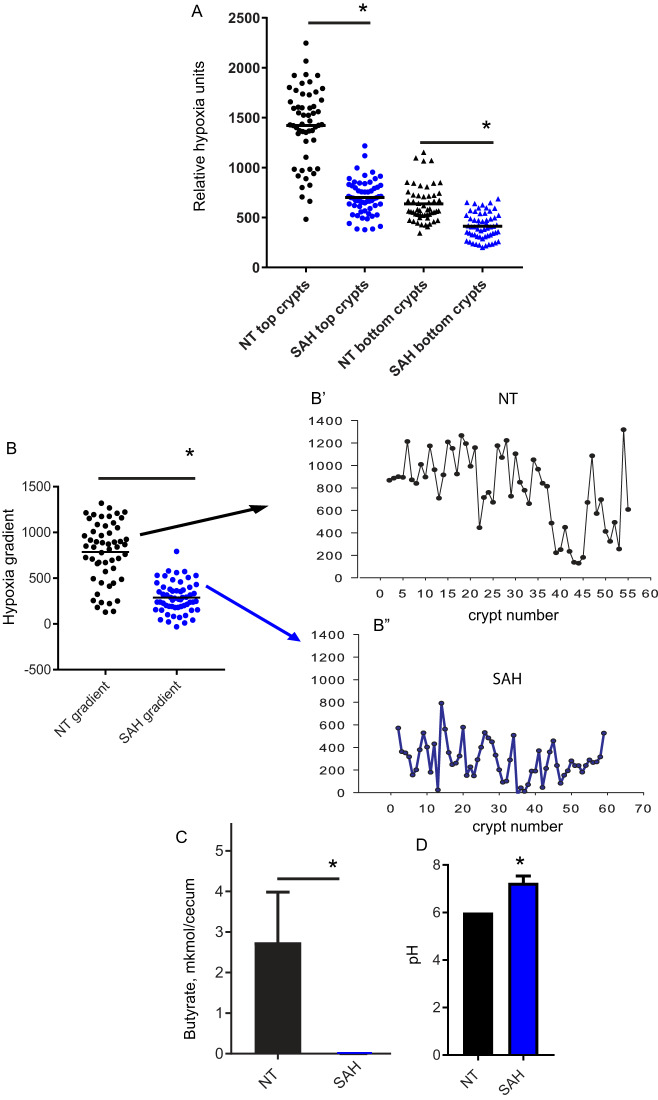
Oxygen, butyrate, and pH are altered following SAH treatment. (A) Hypoxia at the top and bottom of crypts. (B to Bʺ) Hypoxia gradient between top and bottom of crypts, where panel B represents scatterplots in which each dot represents the hypoxia gradient in one crypt whereas panels B′ and Bʺ represent hypoxia gradients in adjacent crypts in the cecum. *, *P* < 0.0001 by Mann-Whitney test. (C) Butyrate extracted from the whole cecum (μmol/cecum). *, *P* < 0.0001 by unpaired two-tailed *t* test. (D) pH in cecal lumen. *, *P* < 0.0001 by unpaired two-tailed *t* test.

The acute loss of cecal microbiota compartmentalization at 24 h after SAH treatment suggested a shift in the physicochemical properties of the cecum. To confirm this, we examined tissue hypoxia, pH, and butyrate concentration, parameters known to influence and be influenced by the community structure, membership, and function of the cecal microbiota (18–21). SAH treatment resulted in a significant increase in oxygen at both top and bottom of the cecal crypt compared to nontreated mice. These findings suggest that the normal hypoxic crypt environment becomes less hypoxic under these conditions (Fig. 9A). Examination of the hypoxia gradient from the top to the bottom of crypts demonstrated that SAH-treated mice displayed a reduced hypoxia gradient (Fig. 9B and B′). Furthermore, the hypoxia gradient was less variable in SAH-treated mice than untreated mice (Fig. 9Bʺ, *P* < 0.001, Levene’s test for equality of variance), suggesting that the changes in the hypoxia imposed by the surgical stress overcome the natural variability. Additionally, SAH treatment led to the depletion of butyrate at nondetectable levels (Fig. 9C) and the luminal pH increase (Fig. 9D) compared with controls, indicating loss of luminal fermentative capacity.

## DISCUSSION

Results from the present study uncover new details on the zonation or compartmentalization pattern between the cecal crypts and lumen based on 16S rRNA gene amplicon and shotgun sequencing of the microbiome. This study demonstrated that under normal conditions, the luminal compartment is dominated by *Bacteroidetes* and *Firmicutes*, which confer digestive functions, whereas the crypts are dominated by *Deferribacteres* and *Proteobacteria*, which confer stress resistance functions. *Deferribacteres* also had a tendency to occupy the crypts within the cecal tip rather than the cecal base, demonstrating that anatomic variability exists within the crypt microbiome. An interesting finding in this study was the contrast in compositional variability between the luminal and crypt microbiome. The observation that the luminal microbiome is less variable in composition within a batch could be due, in part, to the role of the luminal microbiome in the process of digestion in conjunction with the use of a standard chow-based diet in all of our experiments. On the other hand, the variability in crypt microbiome could be due to the uniqueness of the crypt microenvironment which in turn influences microbial composition. One such influence might include the degree of hypoxia at specific locations within crypts given the finding that oxygen was observed to be elevated at the bottom of crypts. It may be for this reason that others have identified aerobes as part of the core crypt microbiome (4).

Surprisingly, we identified the anaerobe *M. schaedleri* as the most abundant microorganism within the cecal crypts in untreated mice. Furthermore, the cooccurrence network analysis demonstrated *M. schaedleri* to be a keystone organism in cecal crypts. The ability of *M. schaedleri* to inhabit cecal crypts may be explained by its specific spiral shape allowing for its mobility in mucus (22). In addition, full-genome analysis of *M. schaedleri* demonstrated that it possesses genes involved in the scavenging of oxygen and reactive oxygen species (23), perhaps explaining how *M. schaedleri* is able to persist at the bottom of crypts where the oxygen concentration is less hypoxic. Finally, *M. schaedleri* possesses genes that encode a type VI secretion system which may allow it to establish either a mutualistic or pathogenic relationship with other bacteria and its host, and putative effector proteins with eukaryote-like domains can be used to carry out specialized interactions with its host (23). Together, these features may allow *M. schaedleri* to predominate and persist at the bottom of crypts as a member of the core cecal crypt microbiome.

The apparent discrepancy between this work defining the anaerobe *M. schaedleri* as a cecal core crypt microbiome member and the work of others demonstrating the aerobe Acinetobacter as a predominant species in the proximal colon core crypt microbiome (4) may be explained by the fact that different intestinal compartments were sampled in each study. Work by Pedron et al. focused its analysis on the proximal colon in mice (4), whereas in the current study, the mouse cecum was analyzed.

Results from the present study demonstrate that, in the process of performing major surgery, i.e., SAH treatment, there is a loss in the lumen-crypt microbiome compartmentalization and loss of function in both compartments. Intriguingly, the depletion of butyrate in response to SAH is similar to the findings when the mouse cecum is surgically removed (2) and suggests a functional impairment of the cecal microbiome postoperatively. When butyrate is deficient, colonocytes are deprived of their key oxidative fuel (24). Butyrate deficiency under these circumstances could lead to a decrease in the hypoxic environment of the intestinal lumen and thus create conditions favorable for facultative anaerobic growth. When the crypt microbiome is perturbed in function, protection against oxidative stress is lost, cross-communication between the crypt microbiome and host cells is impaired, and homeostasis can shift to a pathological state as we previously demonstrated with the stem marker Lgr5 and the proliferation marker Ki67 (7). The increase in crypt oxygenation observed in the present study may explain the disappearance of *M. schaedleri* in mice following SAH treatment. *M. schaedleri* is a major hub for intercompartment signaling. Impairment of microbiome signaling between the lumen, crypt and mucus might result in a bloom of *M. schaedleri* competitors such as *E. aerosaccus* and/or the disappearance of synergistic bacteria such as *Lactobacillus*. The relocation of *E. aerosaccus* from crypt to lumen following SAH treatment and its subsequent propagation within the lumen supports our recent hypothesis that cecal crypts may represent a hidden niche for Proteobacteria propagation and dissemination following surgical stress (25). Additionally, increased oxygenation within the crypts favors oxygen-resistant bacteria to populate the crypts leading to potential colonization by pathogenic bacteria and destruction of the crypts (7).

This study has several limitations. First, we did not examine the individual contributions of starvation, antibiotics, and surgery in this model as we wanted to closely mimic the actual process of performing major surgery that occurs in 95% or more of surgical procedures in the United States, which involves overnight fasting and antibiotic exposure. Second, it is possible that crypt-to-crypt variability in the hypoxia gradient could have contributed to the observed crypt-to-crypt variability in the microbiome, which was not specifically addressed. By using several aliquots of pooled crypts for one sequencing sample, we were not able to detail the differences in crypt-to-crypt microbiome across a specific region of the cecum. Finally, we used a single mouse strain from a single vendor housed within a single animal facility, and therefore, these variables were not accounted for in the analysis.

### Conclusion.

The lumen, mucus, and crypt microbiomes represent distinct communities of bacteria each with their own unique composition. *M. schaedleri* was identified to be a keystone member of the cecal crypt microbiome community, providing functions essential to shaping the crypt microenvironment. The lumen microbiome was characterized by its carbon metabolism whereas the crypt microbiome was characterized by its stress resistance functions.

Compartmentalization of the cecal crypt and lumen microbiome was lost when the host was subjected to provocative conditions such as those encountered during a major surgical procedure. Results of this study provide important observations on the disturbance effect that major surgery and attendant exposures such as overnight fasting and antibiotics has on the composition and function of cecal microbiome. A more complete understanding of this observation may inform how restoration of the spatial context, composition, and function of the cecal microbiome following surgery influences postoperative recovery.

## MATERIALS AND METHODS

### Collection and processing of cecal samples for DNA extraction.

All mouse protocols and experiments described in this study were approved by the Institutional Animal Care and Use Committee and performed in accordance with Institutional Biosafety Committee protocols at the University of Chicago (IACUC protocol 71744, IBC protocol 968). C57/BL6 7- to 8-week-old male mice (Charles River Laboratories, Raleigh, NC, USA) were used in all experiments. Mice were euthanized, and two portions of cecum were collected: the portion most proximal to the ileocecal junction, here defined as the base, and the distal blind end, here defined as the tip (see Fig. S1 in the supplemental material). Collected tissues were cut in small pieces and fixed in PAXgene tissue fix reagent followed by stabilization with PAXgene tissue stabilizer (Qiagen, Valencia, CA, USA). Stabilized samples were embedded in paraffin. Slices of 10 μm in thickness were cut on glass Leica Microsystems PEN MembranSlides (Fisher, Fort Myers, FL, USA). Slides were counterstained (hematoxylin and eosin [H&E]) before laser capture microdissection (LCM). Crypt, mucus layers, and luminal contents were extracted by LCM using a Leica LMD 6500 laser capture microdissector. From each slide, the entire luminal contents were extracted first, then the mucus layer, and finally the crypt contents (Fig. S2). All crypts were visually inspected microscopically, and those containing bacteria were sampled. For 16S rRNA analysis, DNA was extracted from the entire slide of 10 μm in thickness that contained 40 to 70 crypts. For metagenomics analysis, the crypts from 6 slides (approximately 300 crypts) from two mice (∼600 crypts total) were harvested. Luminal and mucus contents were also harvested from the same slides. The contents were collected in Axygen PCR tubes (Axygen, Union City, CA, USA). In each session of LCM, an empty tube was inserted in the tube holder to identify environmental contamination during the collection process. These environmental tubes were subsequently processed along the cryptal, mucus, and luminal samples. The environmental controls were included in each LCM session.

### DNA extraction.

Eighty microliters of lysozyme (Sigma-Aldrich, St. Louis, MO, USA) as a solution of 10 mg/ml in RNase-free phosphate-buffered saline (PBS) (Fisher, Hanover Park, IL, USA) was dropped on the tube cap containing captured content and incubated for 1 h at room temperature (RT). One hundred fifty microliters of proteinase K (Applied Biosystems Arcturus PicoPure DNA extraction kit; Life Technologies, Grand Island, NY, USA) was added and incubated for 24 h at 65°C, and then the reaction was stopped by heating for 10 min at 95°C. DNA was stored at −20°C.

### DNA processing and sequencing.

For 16S rRNA gene amplicon sequencing, PCR amplicon libraries targeting the 16S rRNA encoding gene present in metagenomic DNA were produced using a barcoded primer set adapted for the Illumina MiSeq (26). DNA sequence data were generated using Illumina paired-end sequencing. Specifically, the V4 region of the 16S rRNA gene (515F to 806R) was PCR amplified with region-specific primers that include adapter sequences used in the Illumina flow cell (26, 27). The forward amplification primer also contains a 12-base barcode sequence that supports pooling of up to 2,167 different samples in each lane (26, 27). Each 25-μl PCR mixture contains 9.5 μl of Mo Bio PCR water (certified DNA-free), 12.5 μl of QuantaBio’s AccuStart II PCR ToughMix (2× concentration, 1× final), 1 μl Golay barcode-tagged forward primer (5 μM concentration, 200 pM final), 1 μl reverse primer (5 μM concentration, 200 pM final), and 1 μl of template DNA. The conditions for PCR were as follows: 94°C for 3 min to denature the DNA, with 35 cycles at 94°C for 45 s, 50°C for 60 s, and 72°C for 90 s, and a final extension of 10 min at 72°C to ensure complete amplification. Amplicons were then quantified using PicoGreen (Invitrogen) and pooled into a single tube so that each amplicon was represented in equimolar amounts. This pool was then cleaned up using AMPure XP beads (Beckman Coulter) and subsequently quantified using a fluorometer (Qubit; Invitrogen). After quantification, the molarity of the pool was determined and diluted down to 2 nM, the pool was denatured, and then molarity was further diluted to a final concentration of 6.75 pM with a 10% PhiX spike for sequencing on the Illumina MiSeq. Amplicons were sequenced on a 151-bp by 12-bp by 151-bp MiSeq run using customized sequencing primers and procedures (26). Apart from the LCM extraction controls, an empty blank was employed during each extraction to account for the possible contamination present in the extraction kits and downstream processing. For metagenomics analysis, genomic DNA was quantified using the Invitrogen Qubit and sheared using the Covaris sonicator to the desired size range. Metagenomic shotgun libraries were then generated using TaKaRa’s Apollo324 automated library system and Illumina-compatible PrepX DNA library kits (TaKaRa) according to the manufacturer’s instructions. Shotgun libraries were then sequenced on a 2- by 151-bp lane of the Illumina HiSeq2500. As before, apart from the LCM extraction controls, an empty blank was employed during each extraction to account for the possible contamination present in the extraction kits and downstream processing.

### Sequence identification and filtering.

Identification of the exact sequence variants (ESVs) in each sample was performed employing DADA2 (28), using default parameters unless indicated otherwise. Illumina forward and reverse sequences were split by sample using the QIIME function “*split_sequence_file_on_sample_ids.py*” (29). Subsequently, all the reverse and forward sequences for all four sequencing runs were combined. Seven samples, most of them contamination controls, were removed due to low number of reads (<10 reads). Paired reverse and forward sequences were filtered, and both were truncated to a maximum 150-bp length with maximum expectation of 1 (maxEE). Sequences were demultiplexed, and the error sequencing rates were learned and employed to infer the most likely reverse and forward sequences, which were subsequently merged to create a final sequence table. Only nonchimeric sequences whose lengths were between 251 and 255 bp were used for the downstream analysis (95% of the merged sequences lay within this base pair length range). ESV taxonomy was assigned using Silva database v.132 (30). ESVs present in contamination control samples, including environmental contamination during LCM extraction and extraction kits, were removed from downstream analysis. We subtracted from each ESV five times the maximum reads for that same ESV present in the environmental control samples. Finally, samples whose abundance was less than 1% were further removed. All analyses were done in R (31).

### Identification of intravariability and intervariability in lumen and crypt.

Samples were rarefied to 2,000 reads, and unweighted nonnormalized UniFrac distances (32) were employed to establish whether there was a statistically significant difference as a function of all possible experimental values using permutational multivariate analysis of variance (PERMANOVA). Explored variables included technical replicates, mouse batch, mice within the batch, tissue slides, sequencing run, location within the colon (i.e., base versus tip), and luminal, mucus, and cryptal contents. Permutations to identify sliding differences with PERMANOVA were blocked by cecal contents (e.g., lumen, mucus, and cryptal), batch extraction, sequencing run, and mouse; mice by cecal contents (e.g., lumen, mucus, and cryptal), batch extraction, and sequencing run; extraction and sequencing variability by cecal contents (e.g., lumen, mucus, and cryptal) and colon location (i.e., tip and base); cecal location by cecal contents (e.g., lumen, mucus, and cryptal) and batch extraction and sequencing run; cecal contents by colon location, batch extraction, and sequencing run. Permutations were blocked using the function *setBlocks* from the *phyloseq* package. Calculations were performed using R packages *metagenomeSeq* (33), *phyloseq* (34), and *vegan* (https://CRAN.R-project.org/package=vegan). Graphs were plotted with R package *ggplot* (35).

### Community structure, composition, and network analysis.

α-Diversity differences between the samples were identified using Shannon diversity indexes between cumulative sum scaling (CSS)-normalized samples (33). After the CSS normalization, there were no statistically significant differences between crypt and lumen read counts (see Fig. S3 in the supplemental material). Statistically significant differences in Shannon diversity indexes were estimated by PERMANOVA blocking by extraction batch and sequencing run. β-Diversity differences were deemed significant using PERMANOVA of rarefied samples at 2,000 reads using weighted normalized UniFrac distances. Rarefaction was employed only for calculating phylogenic distances for UniFrac between the samples that were used in UniFrac methods, as UniFrac authors claimed that it provided the best resolution for their methods (36). All the other calculations in the manuscript were done on CSS-normalized data. Rarefaction removed 9 samples, all of them collected from crypts (92% of the samples remained after rarefaction). Those samples had an average number of reads of 1,375, while the rest of the samples had an average total read number of 21,093. In total, we removed 152,714 sequence reads and retained 2,341,399 sequence reads, 97% of the total sequence reads. All β-diversity PERMANOVA calculations were blocked by extraction batch and sequencing run. Phyla and ESV were deemed significant using a zero-inflated, gaussian distribution, mixture-model with posterior-probability weighting of phylum- or ESV-normalized abundances which were normalized using CSS normalization (33). All models were corrected by extraction batch and sequencing run. Phylum or ESV CSS that had a *P* value of ≤0.001 after false-discovery rate (FDR) correction (37) were deemed significant. Coabundance networks between significant ESVs were identified with SparCc (38). Estimation of *P* values was done by *t* tests comparing the average correlation coefficients of each edge in a randomly selected sample of 90% of the data and random shuffling of the same randomly selected sample to remove the data structure. We employed a total of 500 bootstrapping iterations. Edges were considered significant if FDR-corrected *P* value was ≤0.1. Network properties were estimated with R package *igraph* (http://igraph.org). Networks were plotted with Cytoscape (39).

10.1128/mSystems.00377-20.3 FIG S3 Comparison of read counts obtained between crypts and lumen. (A) Number of raw reads (log scale), *P* value < 0.0001. (B) Number of raw reads (log scale) after removing contamination from the samples, *P* value < 0.0001. (C) Number of CSS-normalized reads (log scale), *P* value = 0.06. Download FIG S3, PDF file, 0.3 MB. Copyright © 2020 Zaborin et al. 2020 Zaborin et al. This content is distributed under the terms of the Creative Commons Attribution 4.0 International license.

10.1128/mSystems.00377-20.4 FIG S4 16S rRNA analysis of environmental controls. Contamination tree with detailed hierarchy from 6 distinct runs. Download FIG S4, PDF file, 0.3 MB. Copyright © 2020 Zaborin et al. 2020 Zaborin et al. This content is distributed under the terms of the Creative Commons Attribution 4.0 International license.

10.1128/mSystems.00377-20.5 FIG S5 Correlative analysis of ESV composition within the lumen and the crypts. Download FIG S5, PDF file, 0.1 MB. Copyright © 2020 Zaborin et al. 2020 Zaborin et al. This content is distributed under the terms of the Creative Commons Attribution 4.0 International license.

### Predicted metabolism.

Using the curated database Piphillin (11), we mapped each of the identified significant ESV sequences identified above to the genes that encode enzymes present in each sample and the associated metabolic pathways and biochemical reactions. We employed the Kyoto Encyclopedia of Genes and Genomes (KEGG) October 2018 database (40) and BioCyc 22.5 (41). Enzymes, pathways, and biochemical reactions were deemed significant if FDR-corrected *P* values from the nonparametric Wilcoxon test were less than 0.001 with a confidence interval of 0.99, unless indicated otherwise. Specific metabolic pathways were obtained using the canonical pathways in the KEGG database.

### Metagenomic analysis.

Shotgun sequencing of DNA isolated from LCM-extracted microbiome in crypt and lumen was used for metagenomics analysis. Quality control of the metagenomic reads was conducted as described previously (42). Briefly, raw reads were processed for low-quality-based filtering using the Trimmomatic pipeline (43). Host-derived reads were excluded by mapping the reads to the reference mouse genome (GCA_000001635.8) using BBMap software (sourceforge.net/projects/bbmap/). Quality-trimmed reads were processed for taxonomic and functional profiling using Metaphlan2 (44) and Humann2 (12), respectively. Differential feature selection was performed using Fisher’s exact-*t* test. We assessed the statistical significance (FDR-corrected *P* value < 0.05) for taxon and pathway abundances (based on the genes that encode enzymes) according to sample types. Downstream analysis highlighted that proportions of the host-originated reads are similar in the crypt and luminal samples (i.e., 0.21 to 6%); therefore, we assume no potential bias in our analysis.

### SEM.

The SEM of cecal tissue fixed with 3% glutaraldehyde in 0.1 M phosphate buffer, at pH 7.2 was performed as previously described (7).

### *Mucispirillum* detection by the FISH.

A probe for *Mucispirillum* strain ATO550 (yellow) was synthesized by MetaSystems-Indigo GmbH (Duesseldorf, Germany) using beacon-based technology (45, 46). The specificity of the probe was tested by MetaSystems-Indigo Company.

Fixed paraffin-embedded tissues (FPET) were cut into 5-μm sections, melted at 67°C for 1 h followed by dehydration (xylene, 5 min for 3 times; 100% ethanol, 1 min for 3 times), and then dried on air. The lysis buffer provided by MetaSystems-Indigo was dropped on the tissue slide (10 μl per field), and we waited until it dried. After 5 min for completing dehydration with 100% ethanol, the 10-μl probe was applied followed by hybridization for 1.5 h in the hybridization chamber (Boekel; Advanced Cell Diagnostics). Finally, we incubated the tissue with the stop solution for 1 min, at room temperature (RT). After one more step of dehydration with 100% ethanol for 1 min, at room temperature, the Molecular Probes ProLong Diamond antifade mountant with 4′,6-diamidino-2-phenylindole (DAPI; ThermoFisher Scientific, Eugene, OR) was applied. Confocal microscopy was performed on a Leica SP5 II AOBS tandem scanner spectral confocal system on a DMI6000 microscope and controlled by LASAF software (version 2.8.3).

### Surgical treatment protocol.

Mice were routinely fed tap water and Harland Teklad feed (Madison, WI) under 12-h light/dark cycles and were allowed to acclimate for at least 48 h before surgery. To mimic the major surgical procedure that invariantly includes preoperative starvation and one-dose antibiotic treatment, mice underwent overnight starvation and an intramuscular injection of cefoxitin at a concentration of 25 mg/kg of body weight into the left thigh 30 min prior to surgery. A 30% left lobe hepatectomy was performed via a midline abdominal incision using electrocautery, following which the abdomen was closed and mice were returned to their cages with an average surgical procedure time of 20 min. Mice were euthanized after 1 and 2 days postsurgery (POD1 and POD2, respectively), and cecal samples were collected and processed as described above.

### Measurement of hypoxia in crypts.

Pimonidazole was used as a hypoxia marker (18). We measured hypoxia by immunostaining with a Hypoxyprobe Omni kit that includes pimonidazole hydrochloride, affinity-purified rabbit antipimonidazole antibody (Hypoxyprobe, Inc., Burlington, MA, USA), and goat anti-rabbit IgG (catalog no. A-21428, Alexa Fluor 555; Life Technologies, Grand Island, NY, USA). Pimonidazole was injected intraperitoneally (60 mg/kg) into mice 30 min prior to euthanization. Cecal tissue was cut into small pieces of ∼5 mm and fixed in 10% formalin. Paraffin sections were used for immunostaining with a primary antipimonidazole antibody at 1:50 dilution and secondary goat anti-rabbit antibody at 1:500 dilution.

Confocal images were collected on a Leica TCS SP5 II AOBS confocal system using a DMI6000 microscope, 40× numerical aperture (NA) 1.3 or 100× NA 1.45 oil objective, and LASAF acquisition software. All images were collected with identical laser power and detection settings in a sequential capture to eliminate signal cross talk. Images were analyzed in Fiji (47). A custom macro was designed to measure hypoxia probe mean intensity for hand-drawn regions of interest at the luminal tip and the base of selected crypts. The hypoxia gradient was calculated from these values.

### Butyrate measurement by GC-MS.

Short-chain fatty acids were extracted from mouse cecal contents using diethyl ether (Fisher Scientific), derivatized using *N*-tert-butyldimethylsilyl-*N*-methyltrifluoroacetamide with 1% tert-butyldimethylchlorosilane (Sigma), and run on an Agilent Single Quad gas chromatograph-mass spectroscope (GC-MS) (5977A Single Quad and 7890B GC). An internal standard of 4-methylvaleric acid (277827-5G; Sigma) was used to determine extraction efficiency. We employed the Agilent Mass Hunter qualitative analysis software to extract the ion chromatograms. Area under the curve was calculated and compared to standard curves to quantify the total amount of butyrate present in each sample. All values were normalized to the cecal total mass.

### Data availability.

The raw data from 16S rRNA amplicon sequencing and shotgun sequencing were deposited in the NCBI database under the BioProject ID PRJNA591641.

## References

[1] Laurin M, Everett ML, Parker W 2011 The cecal appendix: one more immune component with a function disturbed by post-industrial culture. Anat Rec (Hoboken) 294:567–579. doi:10.1002/ar.21357. 21370495

[2] Brown K, Abbott DW, Uwiera RRE, Inglis GD 2018 Removal of the cecum affects intestinal fermentation, enteric bacterial community structure, and acute colitis in mice. Gut Microbes 9:218–235. doi:10.1080/19490976.2017.1408763. 29227180 PMC6291264

[3] Chambers ES, Preston T, Frost G, Morrison DJ 2018 Role of gut microbiota-generated short-chain fatty acids in metabolic and cardiovascular health. Curr Nutr Rep 7:198–206. doi:10.1007/s13668-018-0248-8. 30264354 PMC6244749

[4] Pedron T, Mulet C, Dauga C, Frangeul L, Chervaux C, Grompone G, Sansonetti PJ 2012 A crypt-specific core microbiota resides in the mouse colon. mBio 3:e00116-12. doi:10.1128/mBio.00116-12. 22617141 PMC3372965

[5] Mantani Y, Ito E, Nishida M, Yuasa H, Masuda N, Qi WM, Kawano J, Yokoyama T, Hoshi N, Kitagawa H 2015 Ultrastructural study on the morphological changes in indigenous bacteria of mucous layer and chyme throughout the rat intestine. J Vet Med Sci 77:1121–1128. doi:10.1292/jvms.15-0139. 25890991 PMC4591154

[6] Davis CP, Mulcahy D, Takeuchi A, Savage DC 1972 Location and description of spiral-shaped microorganisms in the normal rat cecum. Infect Immun 6:184–192. doi:10.1128/IAI.6.2.184-192.1972. 4120246 PMC422513

[7] Zaborin A, Krezalek M, Hyoju S, Defazio JR, Setia N, Belogortseva N, Bindokas VP, Guo Q, Zaborina O, Alverdy JC 2017 Critical role of microbiota within cecal crypts on the regenerative capacity of the intestinal epithelium following surgical stress. Am J Physiol Gastrointest Liver Physiol 312:G112–G122. doi:10.1152/ajpgi.00294.2016. 27979825 PMC5338606

[8] Sheik CS, Reese BK, Twing KI, Sylvan JB, Grim SL, Schrenk MO, Sogin ML, Colwell FS 2018 Identification and removal of contaminant sequences from ribosomal gene databases: lessons from the census of deep life. Front Microbiol 9:840. doi:10.3389/fmicb.2018.00840. 29780369 PMC5945997

[9] Riva A, Kuzyk O, Forsberg E, Siuzdak G, Pfann C, Herbold C, Daims H, Loy A, Warth B, Berry D 2019 A fiber-deprived diet disturbs the fine-scale spatial architecture of the murine colon microbiome. Nat Commun 10:4366. doi:10.1038/s41467-019-12413-0. 31554820 PMC6761162

[10] Robertson BR, O’Rourke JL, Neilan BA, Vandamme P, On SLW, Fox JG, Lee A 2005 Mucispirillum schaedleri gen. nov., sp. nov., a spiral-shaped bacterium colonizing the mucus layer of the gastrointestinal tract of laboratory rodents. Int J Syst Evol Microbiol 55:1199–1204. doi:10.1099/ijs.0.63472-0. 15879255

[11] Iwai S, Weinmaier T, Schmidt BL, Albertson DG, Poloso NJ, Dabbagh K, DeSantis TZ 2016 Piphillin: improved prediction of metagenomic content by direct inference from human microbiomes. PLoS One 11:e0166104. doi:10.1371/journal.pone.0166104. 27820856 PMC5098786

[12] Franzosa EA, McIver LJ, Rahnavard G, Thompson LR, Schirmer M, Weingart G, Lipson KS, Knight R, Caporaso JG, Segata N, Huttenhower C 2018 Species-level functional profiling of metagenomes and metatranscriptomes. Nat Methods 15:962–968. doi:10.1038/s41592-018-0176-y. 30377376 PMC6235447

[13] Neshich IA, Kiyota E, Arruda P 2013 Genome-wide analysis of lysine catabolism in bacteria reveals new connections with osmotic stress resistance. ISME J 7:2400–2410. doi:10.1038/ismej.2013.123. 23887172 PMC3834855

[14] O’Doherty PJ, Lyons V, Tun NM, Rogers PJ, Bailey TD, Wu MJ 2014 Transcriptomic and biochemical evidence for the role of lysine biosynthesis against linoleic acid hydroperoxide-induced stress in Saccharomyces cerevisiae. Free Radic Res 48:1454–1461. doi:10.3109/10715762.2014.961448. 25184342

[15] Tsuchiya Y, Zhyvoloup A, Bakovic J, Thomas N, Yu BYK, Das S, Orengo C, Newell C, Ward J, Saladino G, Comitani F, Gervasio FL, Malanchuk OM, Khoruzhenko AI, Filonenko V, Peak-Chew SY, Skehel M, Gout I 2018 Protein CoAlation and antioxidant function of coenzyme A in prokaryotic cells. Biochem J 475:1909–1937. doi:10.1042/BCJ20180043. 29626155 PMC5989533

[16] Busch AW, Montgomery BL 2015 Interdependence of tetrapyrrole metabolism, the generation of oxidative stress and the mitigative oxidative stress response. Redox Biol 4:260–271. doi:10.1016/j.redox.2015.01.010. 25618582 PMC4315935

[17] Dailey HA, Dailey TA, Gerdes S, Jahn D, Jahn M, O’Brian MR, Warren MJ 2017 Prokaryotic heme biosynthesis: multiple pathways to a common essential product. Microbiol Mol Biol Rev 81:e00048-16. doi:10.1128/MMBR.00048-16. 28123057 PMC5312243

[18] Zheng L, Kelly CJ, Colgan SP 2015 Physiologic hypoxia and oxygen homeostasis in the healthy intestine. A review in the theme: cellular responses to hypoxia. Am J Physiol Cell Physiol 309:C350–360. doi:10.1152/ajpcell.00191.2015. 26179603 PMC4572369

[19] Baxter NT, Schmidt AW, Venkataraman A, Kim KS, Waldron C, Schmidt TM 2019 Dynamics of human gut microbiota and short-chain fatty acids in response to dietary interventions with three fermentable fibers. mBio 10:e02566-18. doi:10.1128/mBio.02566-18. 30696735 PMC6355990

[20] Zhai S, Qin S, Li L, Zhu L, Zou Z, Wang L 2019 Dietary butyrate suppresses inflammation through modulating gut microbiota in high-fat diet-fed mice. FEMS Microbiol Lett 366:fnz153. doi:10.1093/femsle/fnz153. 31295342

[21] Hansen TH, Thomassen MT, Madsen ML, Kern T, Bak EG, Kashani A, Allin KH, Hansen T, Pedersen O 2018 The effect of drinking water pH on the human gut microbiota and glucose regulation: results of a randomized controlled cross-over intervention. Sci Rep 8:16626. doi:10.1038/s41598-018-34761-5. 30413727 PMC6226457

[22] Swidsinski A, Sydora BC, Doerffel Y, Loening-Baucke V, Vaneechoutte M, Lupicki M, Scholze J, Lochs H, Dieleman LA 2007 Viscosity gradient within the mucus layer determines the mucosal barrier function and the spatial organization of the intestinal microbiota. Inflamm Bowel Dis 13:963–970. doi:10.1002/ibd.20163. 17455202

[23] Loy A, Pfann C, Steinberger M, Hanson B, Herp S, Brugiroux S, Gomes Neto JC, Boekschoten MV, Schwab C, Urich T, Ramer-Tait AE, Rattei T, Stecher B, Berry D 2017 Lifestyle and horizontal gene transfer-mediated evolution of *Mucispirillum schaedleri*, a core member of the murine gut microbiota. mSystems 2:e00171-16. doi:10.1128/mSystems.00171-16. 28168224 PMC5285517

[24] Byndloss MX, Olsan EE, Rivera-Chávez F, Tiffany CR, Cevallos SA, Lokken KL, Torres TP, Byndloss AJ, Faber F, Gao Y, Litvak Y, Lopez CA, Xu G, Napoli E, Giulivi C, Tsolis RM, Revzin A, Lebrilla CB, Bäumler AJ 2017 Microbiota-activated PPAR-gamma signaling inhibits dysbiotic Enterobacteriaceae expansion. Science 357:570–575. doi:10.1126/science.aam9949. 28798125 PMC5642957

[25] Hyoju SK, Zaborin A, Keskey R, Sharma A, Arnold W, van den Berg F, Kim SM, Gottel N, Bethel C, Charnot-Katsikas A, Jianxin P, Adriaansens C, Papazian E, Gilbert JA, Zaborina O, Alverdy JC 2019 Mice fed an obesogenic Western diet, administered antibiotics, and subjected to a sterile surgical procedure develop lethal septicemia with multidrug-resistant pathobionts. mBio 10:e00903-19. doi:10.1128/mBio.00903-19. 31363025 PMC6667615

[26] Caporaso JG, Lauber CL, Walters WA, Berg-Lyons D, Huntley J, Fierer N, Owens SM, Betley J, Fraser L, Bauer M, Gormley N, Gilbert JA, Smith G, Knight R 2012 Ultra-high-throughput microbial community analysis on the Illumina HiSeq and MiSeq platforms. ISME J 6:1621–1624. doi:10.1038/ismej.2012.8. 22402401 PMC3400413

[27] Caporaso JG, Lauber CL, Walters WA, Berg-Lyons D, Lozupone CA, Turnbaugh PJ, Fierer N, Knight R 2011 Global patterns of 16S rRNA diversity at a depth of millions of sequences per sample. Proc Natl Acad Sci U S A 108 (Suppl 1):4516–4522. doi:10.1073/pnas.1000080107. 20534432 PMC3063599

[28] Callahan BJ, McMurdie PJ, Rosen MJ, Han AW, Johnson AJA, Holmes SP 2016 DADA2: high-resolution sample inference from Illumina amplicon data. Nat Methods 13:581–583. doi:10.1038/nmeth.3869. 27214047 PMC4927377

[29] Caporaso JG, Kuczynski J, Stombaugh J, Bittinger K, Bushman FD, Costello EK, Fierer N, Peña AG, Goodrich JK, Gordon JI, Huttley GA, Kelley ST, Knights D, Koenig JE, Ley RE, Lozupone CA, McDonald D, Muegge BD, Pirrung M, Reeder J, Sevinsky JR, Turnbaugh PJ, Walters WA, Widmann J, Yatsunenko T, Zaneveld J, Knight R 2010 QIIME allows analysis of high-throughput community sequencing data. Nat Methods 7:335–336. doi:10.1038/nmeth.f.303. 20383131 PMC3156573

[30] Yarza P, Yilmaz P, Pruesse E, Glöckner FO, Ludwig W, Schleifer K-H, Whitman WB, Euzéby J, Amann R, Rosselló-Móra R 2014 Uniting the classification of cultured and uncultured bacteria and archaea using 16S rRNA gene sequences. Nat Rev Microbiol 12:635–645. doi:10.1038/nrmicro3330. 25118885

[31] Kim S 2015 ppcor: an R package for a fast calculation to semi-partial correlation coefficients. Commun Stat Appl Methods 22:665–674. doi:10.5351/CSAM.2015.22.6.665. 26688802 PMC4681537

[32] Lozupone C, Knight R 2005 UniFrac: a new phylogenetic method for comparing microbial communities. Appl Environ Microbiol 71:8228–8235. doi:10.1128/AEM.71.12.8228-8235.2005. 16332807 PMC1317376

[33] Paulson JN, Stine OC, Bravo HC, Pop M 2013 Differential abundance analysis for microbial marker-gene surveys. Nat Methods 10:1200–1202. doi:10.1038/nmeth.2658. 24076764 PMC4010126

[34] McMurdie PJ, Holmes S 2013 phyloseq: an R package for reproducible interactive analysis and graphics of microbiome census data. PLoS One 8:e61217. doi:10.1371/journal.pone.0061217. 23630581 PMC3632530

[35] Wickham H 2016 ggplot2: elegant graphics for data analysis. Springer-Verlag, New York, NY.

[36] Weiss S, Xu ZZ, Peddada S, Amir A, Bittinger K, Gonzalez A, Lozupone C, Zaneveld JR, Vazquez-Baeza Y, Birmingham A, Hyde ER, Knight R 2017 Normalization and microbial differential abundance strategies depend upon data characteristics. Microbiome 5:27. doi:10.1186/s40168-017-0237-y. 28253908 PMC5335496

[37] Benjamini Y, Drai D, Elmer G, Kafkafi N, Golani I 2001 Controlling the false discovery rate in behavior genetics research. Behav Brain Res 125:279–284. doi:10.1016/s0166-4328(01)00297-2. 11682119

[38] Friedman J, Alm EJ 2012 Inferring correlation networks from genomic survey data. PLoS Comput Biol 8:e1002687. doi:10.1371/journal.pcbi.1002687. 23028285 PMC3447976

[39] Shannon P, Markiel A, Ozier O, Baliga NS, Wang JT, Ramage D, Amin N, Schwikowski B, Ideker T 2003 Cytoscape: a software environment for integrated models of biomolecular interaction networks. Genome Res 13:2498–2504. doi:10.1101/gr.1239303. 14597658 PMC403769

[40] Kanehisa M, Goto S 2000 KEGG: Kyoto encyclopedia of genes and genomes. Nucleic Acids Res 28:27–30. doi:10.1093/nar/28.1.27. 10592173 PMC102409

[41] Romero PR, Karp PD 2004 Using functional and organizational information to improve genome-wide computational prediction of transcription units on pathway-genome databases. Bioinformatics 20:709–717. doi:10.1093/bioinformatics/btg471. 14751985

[42] Sangwan N, Zarraonaindia I, Hampton-Marcell JT, Ssegane H, Eshoo TW, Rijal G, Negri MC, Gilbert JA 2016 Differential functional constraints cause strain-level endemism in polynucleobacter populations. mSystems 1:e00003-16. doi:10.1128/mSystems.00003-16. PMC5069759 27822527

[43] Bolger AM, Lohse M, Usadel B 2014 Trimmomatic: a flexible trimmer for Illumina sequence data. Bioinformatics 30:2114–2120. doi:10.1093/bioinformatics/btu170. 24695404 PMC4103590

[44] Segata N, Waldron L, Ballarini A, Narasimhan V, Jousson O, Huttenhower C 2012 Metagenomic microbial community profiling using unique clade-specific marker genes. Nat Methods 9:811–814. doi:10.1038/nmeth.2066. 22688413 PMC3443552

[45] Sakarikou C, Parisato M, Lo Cascio G, Fontana C 2014 Beacon-based (bbFISH(R)) technology for rapid pathogens identification in blood cultures. BMC Microbiol 14:99. doi:10.1186/1471-2180-14-99. 24750976 PMC3997747

[46] Tyagi S, Kramer FR 1996 Molecular beacons: probes that fluoresce upon hybridization. Nat Biotechnol 14:303–308. doi:10.1038/nbt0396-303. 9630890

[47] Schindelin J, Arganda-Carreras I, Frise E, Kaynig V, Longair M, Pietzsch T, Preibisch S, Rueden C, Saalfeld S, Schmid B, Tinevez JY, White DJ, Hartenstein V, Eliceiri K, Tomancak P, Cardona A 2012 Fiji: an open-source platform for biological-image analysis. Nat Methods 9:676–682. doi:10.1038/nmeth.2019. 22743772 PMC3855844

