# Supplementary figures and images for: Spatial Compartmentalization of the Microbiome between the Lumen and Crypts Is Lost in the Murine Cecum following the Process of Surgery, Including Overnight Fasting and Exposure to Antibiotics
Source: mSystems. 2020 Jun 9;5(3):e00377-20. doi: 10.1128/mSystems.00377-20 (PMC7289591; doi:10.1128/mSystems.00377-20)

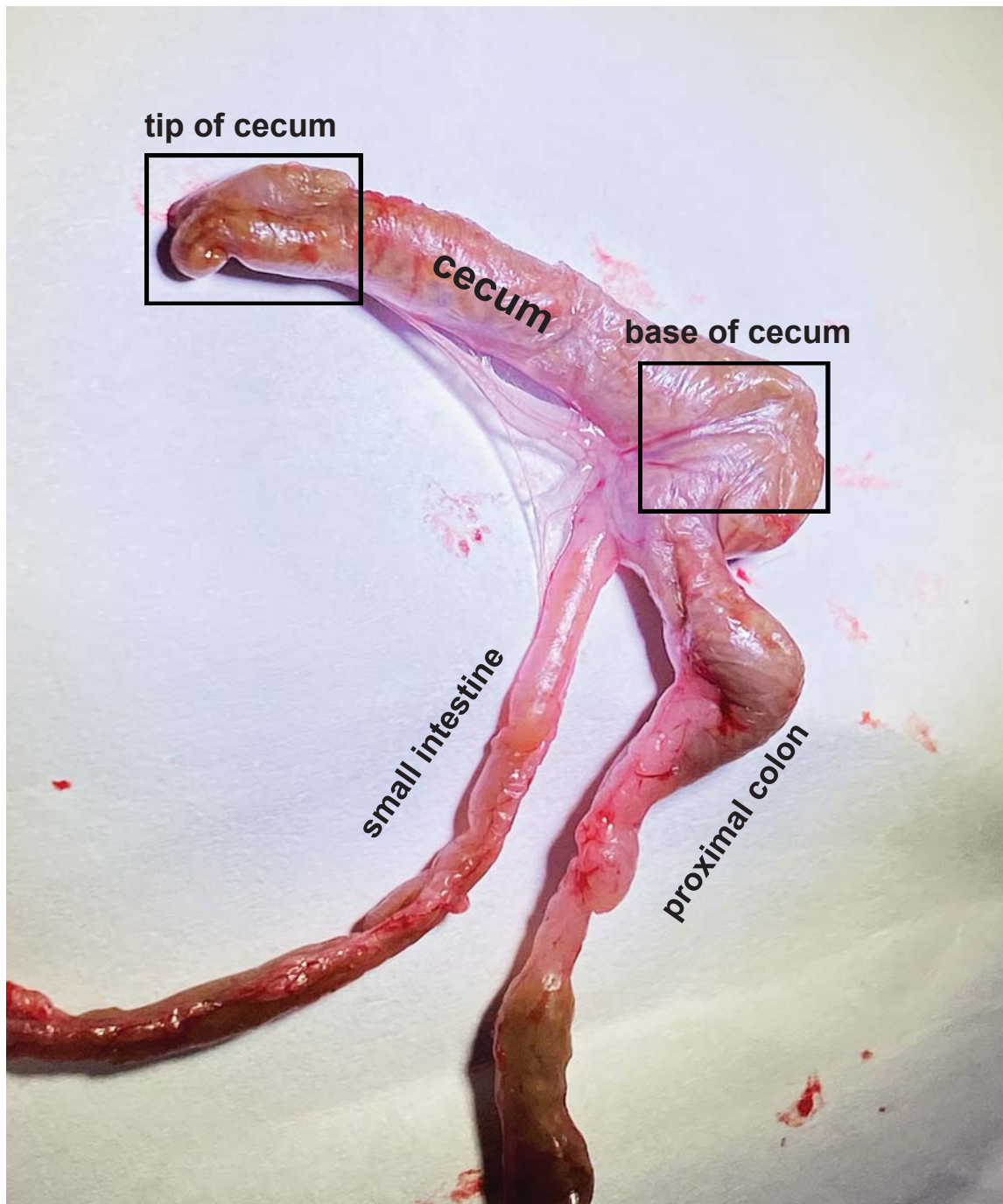

Supplement: FIG S1 [file mSystems.00377-20-sf001.pdf]

Prior to LCM

A

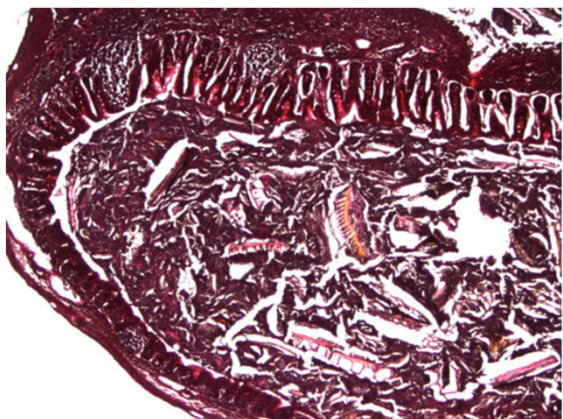

Luminal content extracted

B

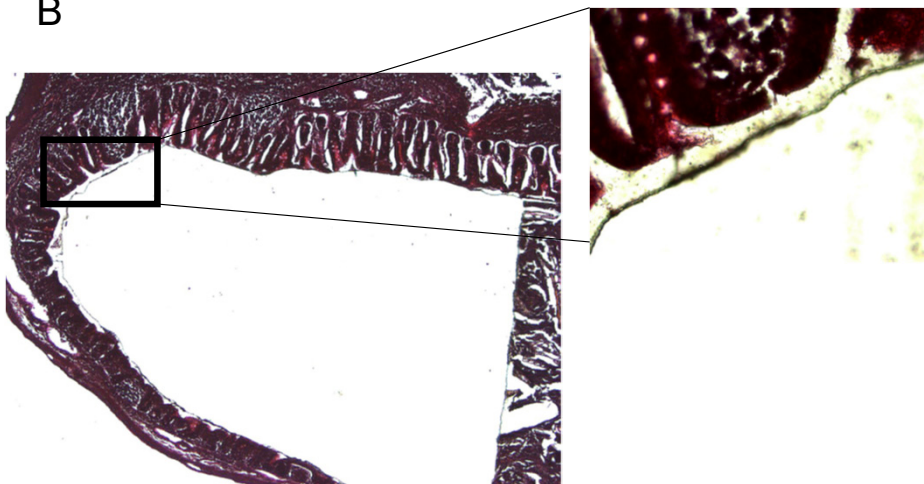

Mucus extracted

C

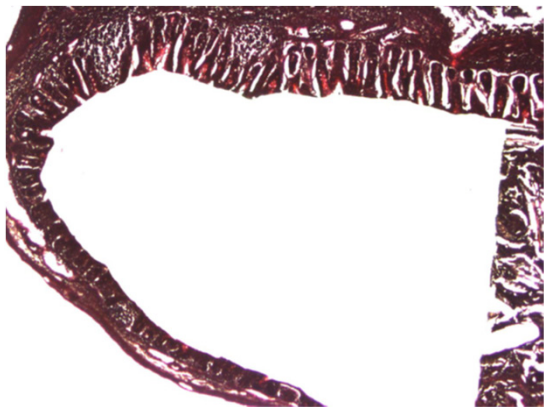

Crypts extracted

D

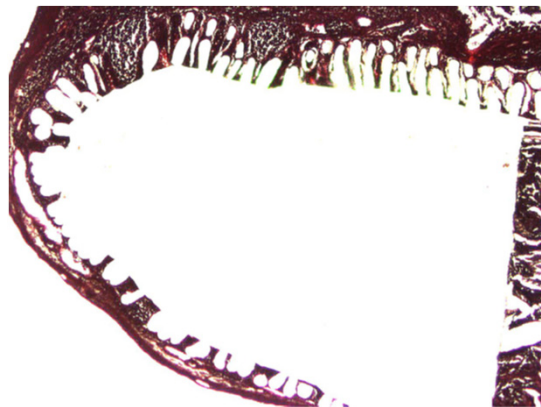

Supplement: FIG S2 [file mSystems.00377-20-sf002.pdf]

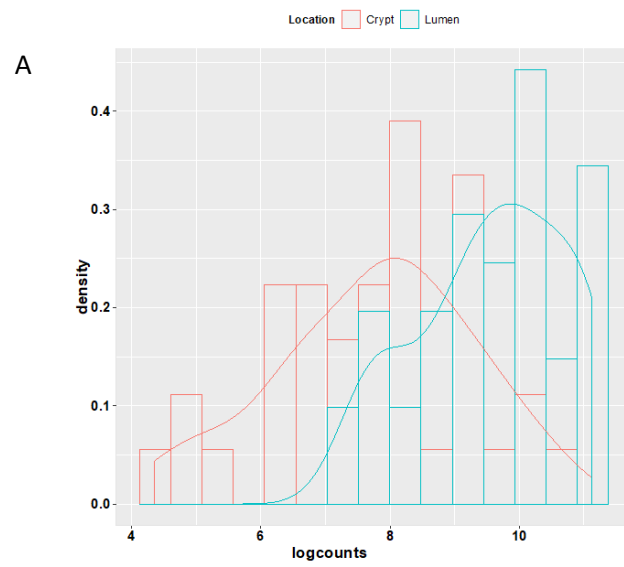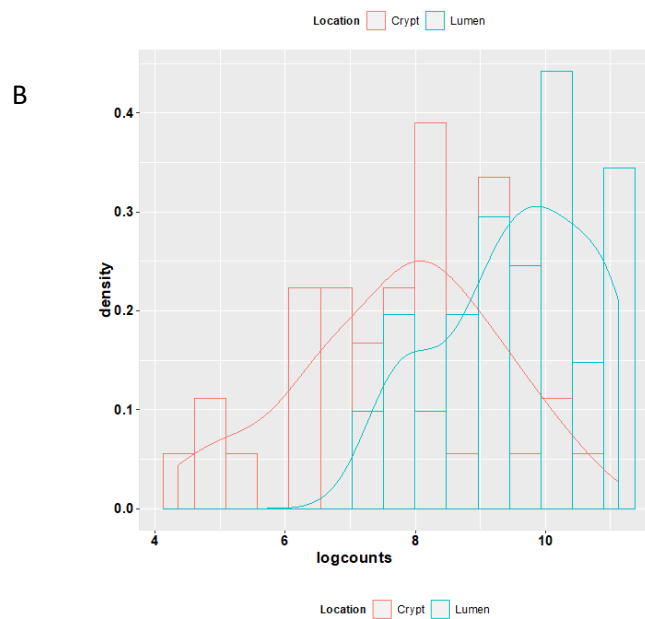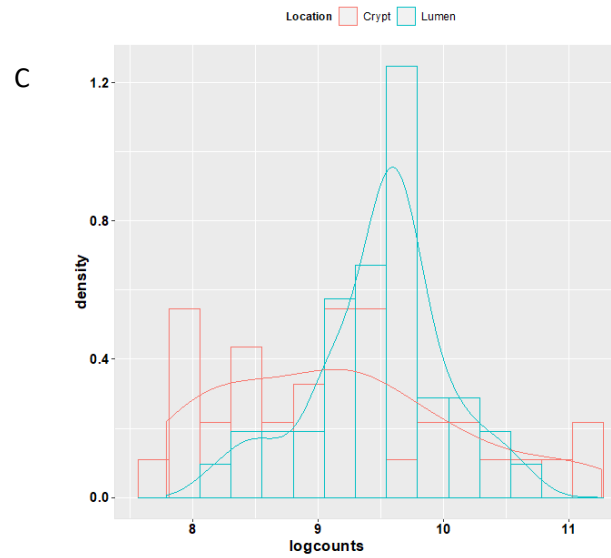

Supplement: FIG S3 [file mSystems.00377-20-sf003.pdf]

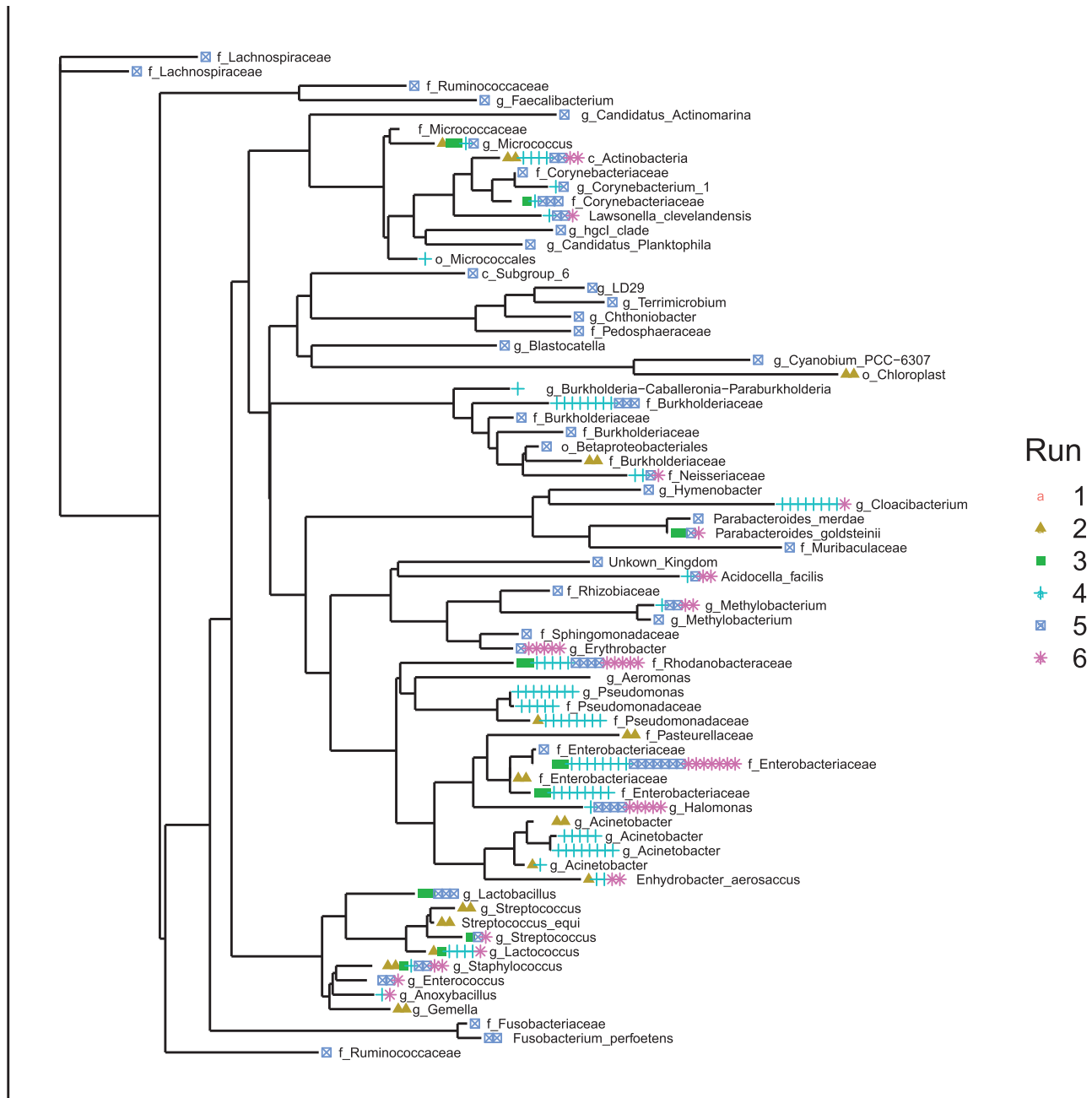

Supplement: FIG S4 [file mSystems.00377-20-sf004.pdf]

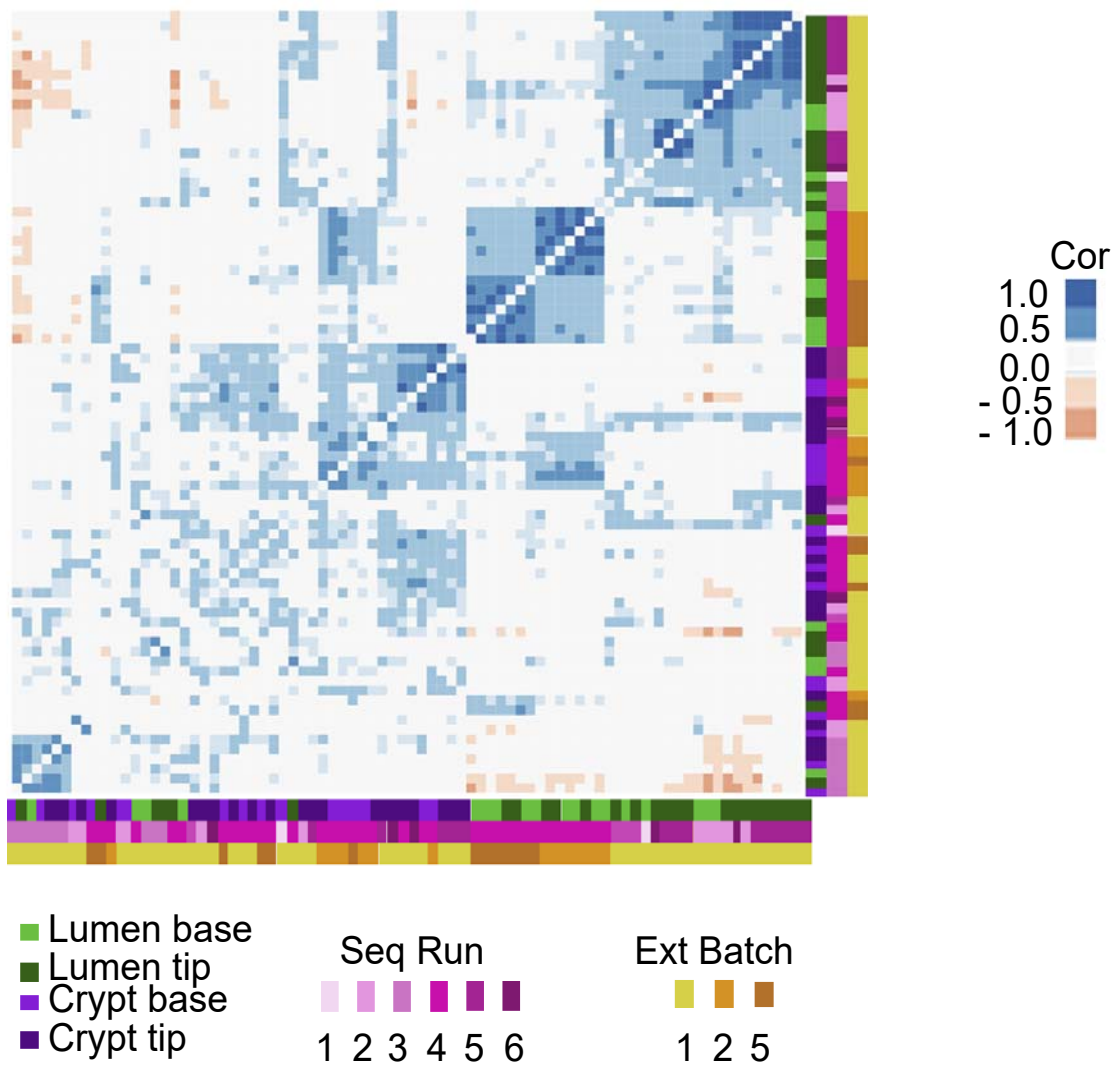

Supplement: FIG S5 [file mSystems.00377-20-sf005.pdf]
