# Supplementary material for: Spatial Compartmentalization of the Microbiome between the Lumen and Crypts Is Lost in the Murine Cecum following the Process of Surgery, Including Overnight Fasting and Exposure to Antibiotics
Source: mSystems. 2020 Jun 9;5(3):e00377-20. doi: 10.1128/mSystems.00377-20 (PMC7289591; doi:10.1128/mSystems.00377-20)
Supplement: TABLE S1 [file mSystems.00377-20-st001.docx]

|  |  |  |  | Crypt | | Lumen | | Samples | |
| --- | --- | --- | --- | --- | --- | --- | --- | --- | --- |
| Run | Batch | Treatment | Mice number | Cecum  Tip | Cecum Base | Cecum  Tip | Cecum  Base | Control | Mouse |
| 1 | 1 | NT | 1 |  | 1 |  | 1 | 2 | 2 |
| 2 | 1 | NT | 4 | 4 | 4 | 4 | 4 | 2 | 16 |
| 3 | 1 | NT | 2* | 2 | 2 | 2 | 2 | 10 | 8 |
|  |  | SAHPOD1 | 3* | 3 | 2 | 3 | 2 |  | 10 |
|  |  | SAHPOD2 | 3* | 2 | 3 | 2 | 3 |  | 10 |
| 4 | 1 | NT | 2 | 2 | 2 | 2 | 2 | 2 | 8 |
|  | 2 | NT | 4 | 3 | 4 | 3 | 4 | 2 | 14 |
|  | 3 | NT | 4 | 4 | 4 | 4 | 4 | 4 | 16 |
|  | 5 | NT | 5 | 4 | 5 | 4 | 5 | 5 | 18 |
| 5 | 1 | NT | 2 | 6 |  | 9 |  | 7 | 21 |
| 6 | 1 | NT | 1 | 3 |  | 2 |  | 10 | 7 |
|  |  |  |  |  |  |  |  |  |  |
|  |  |  |  |  |  |  |  |  |  |
