# Supplementary material for: Spatial Compartmentalization of the Microbiome between the Lumen and Crypts Is Lost in the Murine Cecum following the Process of Surgery, Including Overnight Fasting and Exposure to Antibiotics
Source: mSystems. 2020 Jun 9;5(3):e00377-20. doi: 10.1128/mSystems.00377-20 (PMC7289591; doi:10.1128/mSystems.00377-20)
Supplement: TABLE S3 [file mSystems.00377-20-st003.docx]

| **Source** | **Variable in model** | **p value** | **p adj** |
| --- | --- | --- | --- |
| Technical replicates | Replicate Number | 6.7E-01 | 6.9E-01 |
| Mouse | Mouse Number | 1.4E-01 | 1.7E-01 |
| Run_Batch | RunBatch | 5.0E-04 | 1.4E-03 |
| Cecum base vs cecum tip | Regional changes | 6.9E-01 | 6.9E-01 |
| Lumen vs crypt | Spatial changes | 5.0E-04 | 1.4E-03 |
| Mucus vs crypt | Spatial changes | 1.5E-02 | 2.5E-02 |
| Mucus vs lumen | Spatial changes | 5.0E-04 | 1.4E-03 |
